# Composition and Fatty Acid Profile of Goat Meat Sausages with Added Rice Bran

**DOI:** 10.1155/2014/686298

**Published:** 2014-04-16

**Authors:** Fatemeh Malekian, Margarita Khachaturyan, Sebhatu Gebrelul, James F. Henson

**Affiliations:** Southern University Agricultural Research and Extension Center, P.O. Box 10010, Baton Rouge, LA 70813, USA

## Abstract

A scientific consensus on the relationship between obesity, obesity related diseases, and diet has emerged. One of the factors is overconsumption of the red meats such as pork and beef. Goat meat has the potential to replace these traditionally consumed meats. Rice bran is a rich source of antioxidants such as vitamin E. In this study, goat meat sausages were formulated to contain 0, 1.5 or 3 percent stabilized rice bran. Proximate and fatty acid composition, *α*-tocopherol, cholesterol concentration, and antioxidant activities of cooked goat meat sausages containing varying percentages of rice bran were measured. Data were analyzed using a fixed effects model. The fat percentage in the goat meat sausages increased in response to increasing rice bran percentages (*P* < 0.001). Saturated fatty acids concentration decreased linearly (*P* < 0.01), while unsaturated fatty acids and omega-3 and omega-6 fatty acids increased linearly in response to increasing rice bran percentages (*P* < 0.05). The concentration of *α*-tocopherol in sausages increased linearly in response to increasing rice bran percentages (*P* < 0.01). Also, antioxidant activity increased linearly in sausages in response to added rice bran (*P* < 0.01). The cholesterol concentration of sausages did not vary significantly in response to added rice bran.

## 1. Introduction

A scientific consensus on the relationship between diet and obesity related diseases such as diabetes, heart disease, stroke, and some forms of cancer has been documented [1]. The obesity rate in Louisiana is 28.9%, which is the eighth highest in the USA. Furthermore, Louisiana is in the top seven states in obesity-related medical expenditure [2]. Foods from animal sources have been a part of human diets for years. Even though the percentage of fat in meat has decreased in recent years, the polyunsaturated to saturated fatty acid ratio in meat is usually less than 0.2 when the fat content is above 2% [3]. Blood cholesterol level depends less on the intake of cholesterol from foods and more on the amount of saturated fats consumed, especially the ratio of polyunsaturated to saturated fats [4]. A study has shown that with proper dietary intervention, the mean blood cholesterol level of 90% of a test population was reduced by 3% to 23% [5]. Dietary habits are major factor in the development of obesity and cardiovascular heart diseases.

During the past few decades, epidemiological studies have suggested that a healthy diet and lifestyle are critical for the prevention of many diseases such as breast cancer. Breast cancer is one of the most common cancers and is the second leading cause of death in women. Dietary fat is one of the most studied factors. The most promising dietary fatty acids to reduce the risk of breast cancer are omega-3 polyunsaturated fatty acids [1].

Lean goat meat is low in fat and saturated fatty acids, but high in unsaturated fatty acids such as linoleic and oleic that have been shown to possess hypocholesteremic properties [6, 7]. The chemical composition of goat meat is as follows: moisture 74.2–76.0%; protein 20.6–22.3%; fat 0.6–2.6%; ash 1.1% [8]. Goat meat cuts have protein levels comparable to similarly prepared beef, lamb, and veal but have lower fat content [9]. In addition, the percentage of saturated fat in goat meat is lower than in chicken, beef, pork, or lamb [10, 11].

Considering its high nutritional value and its greater unsaturated to saturated fatty acid ratio, goat meat has the potential to improve the health of susceptible populations without taking meat products out of their daily diet. Consumption of goat meat is becoming popular and is often available at the fine dining level [12].

Currently, there is an increased interest in the use of dietary antioxidants, including vitamins C and E, to prevent cardiovascular diseases [13]. There are many natural antioxidants which can be added to the meat products to increase their health benefits. Some examples are rosemary oil [14], rice bran oil [15], rice bran oil plus rice bran fiber [16], oat bran [6], and oat flour [17]. A number of studies have shown that vitamin E, as a chain-breaking antioxidant, prevents the propagation of free radical reactions [18–20]. Vitamin E inhibits lipid peroxidation* in vitro* and* in vivo* because of its radical scavenging and antioxidant properties [21].

As a major isoform of Vitamin E, *α*-tocopherol is effective* in vivo* only against free radical-mediated oxidation. It inhibits LDL oxidation* in vitro* and has therapeutic effects against atherosclerosis [22]. The rice bran oil antioxidants are very efficient in reducing low density lipoprotein (LDL) and total serum cholesterol [23]. Rice bran oil contains about 0.1–0.14% vitamin E components and 0.9–2.9% oryzanol. The concentrations can vary substantially according to the origin of the rice bran [24, 25]. Vitamin E is a generic term for a group of four tocopherols (*α*-, *β*-, *γ*- and Δ-) and four tocotrienols (*α*-, *β*-, *γ*- and Δ-), of which *α*-tocopherol has the highest biological activity [26].

Rice bran, a byproduct of the rice milling process, is a naturally rich source of antioxidants along with vitamins and minerals. Additionally, it contains 14–16% protein without gluten, 12–23% fat, and 8–10% crude fiber [27]. Nutritional studies in animals and humans have demonstrated the cholesterol lowering potential of rice bran and rice bran fractions [28, 29]. Rice bran fractions such as rice bran oil and soluble fiber are crucial factors in the regulation of plasma cholesterol levels, and the insoluble fiber plays an important role in intestinal regulation [23, 30]. Rice bran also has some beneficial dietary components. These components lowered blood pressure and improved the lipid profile by decreasing the low density lipoprotein (LDL) level in hypertensive rats [31]. Substituting saturated fatty acids in the diet with unsaturated fatty acids such as oleic, linoleic, and linolenic acid lowered low-density lipoprotein-cholesterol in human subjects [32]. The protein in rice bran does not contain gluten, therefore, it is a healthy food choice for people with celiac disease. Approximately, 1 in 133 people in the United States has celiac disease, an immune-mediated disorder associated with gluten, a protein present in wheat, barley, and rye. Most affected individuals experience a remission of the disease after excluding gluten from their diets [33]. In June 2008, the United States Department of Agriculture Food Safety and Inspection Service (USDA FSIS) approved the use of stabilized rice bran as a binder to a maximum of 3.5% in various meat products such as sausage, meatballs, meat loaf, and meat patties [34]. Stabilized rice bran can hold moisture up to three times its weight which can contribute to the juiciness of the goat meat products. Rice bran also has a meat-like texture when it is cooked [35].

The goal of this study was to formulate a healthy goat meat sausage with a higher level of antioxidant activity, a higher level of unsaturated fatty acids, and a lower fat content compared to beef sausages as well as a gluten free product by incorporating varying amounts of stabilized rice bran to the formulation. Nutritional characteristics of raw and cooked goat meat sausages with added rice bran were determined by analyzing the samples for proximate composition and cooked goat meat sausage for fatty acid composition, *α*-tocopherol and cholesterol concentration, and antioxidant activity.

## 2. Materials and Methods

### 2.1. Preparation of Sausages

Goats were reared at the Southern University Agricultural Research and Extension Center (SUAREC) goat farm until slaughter and preparation of products. All meat was prepared in the state inspected Southern University Meat Processing Laboratory. Shoulder, leg, and other parts of one-to-three-year-old goats were deboned and ground.

Rice bran was obtained from Planters Rice Mill in Abbeville, LA and stabilized using microwave heat [36]. The stabilized rice bran was sieved with a 20 mesh screen in order to remove broken rice and husks and to obtain a uniform particle size.

Chili seasoning was obtained from Symrise Inc., Teterboro, NJ. Chili seasoning (3% by weight), salt (1.6% by weight), and water (3.3% by weight) were incorporated into the ground goat meat. Chili seasoning and salt were added for taste, rice bran was added as a binder, and water was added to provide moisture in the mix. The meat samples were divided into three groups. Group 1 was the control with 0%, group 2 with 1.5%, and group 3 with 3% (by weight) stabilized rice bran. The meat/rice bran mixtures were packed into collagen casing. Raw sausages were cooked in a preheated (53.3°C) “Koch” stainless steel cooker/smoker (Koch Supply, North Kansas city, MO). The temperature was increased manually at 2.7°C per hour until the temperature of the cooker reached 65.5°C. The smoker was then turned on and the temperature increased until the internal temperature of the sausages reached 70°C for at least 15 seconds. The cooking and smoking time was 6 to 7 hours. Approximately, 500 g of each raw and cooked sausage were homogenized in a Robot Coup R2 food processor (Robot Coup USA Inc., Ridgeland, MS) for two minutes to obtain a homogeneous sample. Three aliquots were prepared for the raw and cooked sausages and were frozen at −20°C until further analysis.

### 2.2. Proximate Analysis

On the day of analysis, samples were taken out of the freezer early in the morning and were thawed at room temperature. Samples were analyzed using standard American Official of Analytical Chemists (AOAC) approved methods (983.23, 992.15, 920.15, and 985.14) with modifications [37]. Lipids were extracted using a chloroform/methanol solution with butylated hydroxytoluene (BHT), as an antioxidant, and determined gravimetrically. Aliquots were taken from lipid extracts for determination of fatty acids. Protein content was determined using thermal conductivity on a Series II Nitrogen Analyzer 2410 (PerkinElmer Instruments, Norwalk, CT). Ash content was determined with using a Phoenix microwave furnace (CEM corp., Matthews, NC). Moisture content was determined using a Smart system 5 (CEM corp., Matthews, CT). Percentage of carbohydrate was determined by difference using formula: %Carbohydrate = 100 − (%Moisture + %Fat + %Ash + %Proteins). Each sample was run in triplicate.

### 2.3. Fatty Acid Determination

Lipid extracts aliquots (prepared during total lipid extraction according to Section 2.2) were used for the determination of fatty acid composition using a Varian Saturn 2100 (Agilent Technologies Inc. Wilmington DE) Gas Chromatography/Mass Spectrophotometer (GC/MS) with a fused silica column (30 × 0.25 mm) [38]. GLC-490 Reference Standard and C23:0 methyl esters (Internal Standard) were purchased from Nu-Check Prep. Inc. (Elysian, Minnesota, USA) and used for determination of fatty acids. Each sample was run in triplicate.

### 2.4. Simultaneous Determination of *α*-Tocopherol and Cholesterol

Cholesterol and *α*-tocopherol were measured simultaneously on an 1100 Agilent High Performance Liquid Chromatography (HPLC) system (Agilent Technologies Inc. Wilmington DE). Homogenized sausage samples were prepared, extracted and quantified [39]. A ZORBAX RX–SIL 5 µm, 4.8 × 250 mm column was used. The mobile phase was composed of 99% hexane and 1% isopropyl alcohol (HPLC grade) at a flow rate of 0.5 mL/min. The total running time was 12 minutes. The detector was a diode array detector (DAD) operating at *λ* = 202 nm. The injection volume was 5 µL with a needle cleansing system and thermostat temperature of 20°C. The *α*-tocopherol and cholesterol standards and other chemicals were purchased from Sigma Aldrich Co. (St. Louis, MO). Standards were dissolved in hexane. Solutions with 500 ppm of each standard were diluted volumetrically. A calibration curve for each component was made. Analytes were detected using DAD and peak identification which was accomplished by comparing the retention times and HPLC peaks with those obtained from standard solution of mixture analyzed under the same conditions. Quantitative determination was performed using the standard curve. Each sample was run in triplicate.

### 2.5. Antioxidant Activity Determination

The DPPH (2,2-diphenyl-1-picrylhydrazyl) free radical scavenging method was used to measure the antioxidant activity [40] with a slight modification. The chloroform/methanol lipid extraction solution without BHT was used for extraction of components having antioxidant activity. Absorption of the intermediate colored complex was measured on UV-Vis Beckman Coulter spectrophotometer (Loveland, Colorado) at 515 nm and the results were expressed in terms of micromole equivalents of Trolox (6-hydroxy-2,5,7,8-tetramethylchroman-2-carboxylic acid) per 100 grams of sample [40]. The DPPH radical and Trolox were purchased from Sigma Aldrich Co. (St. Louis, MO). Each sample was run in triplicate.

### 2.6. Experimental Design and Statistical Analysis

The study was conducted as a two-factor experiment in a completely randomized design with three replicates. The two factors were sausage preparation (raw or cooked) and three levels of rice bran inclusion (0, 1.5 and 3%). The experimental data were analyzed using PROC GLM of SAS version 9.3 [41]. The data were fitted to a fixed model with the factors sausage preparation and rice bran level treated as fixed effects. The treatment means were compared using the least squares means method of PROC GLM. The effects of the rice bran percentages were compared with trend analysis using orthogonal polynomial contrasts.

## 3. Results and Discussion

### 3.1. Proximate Analyses

The results of the proximate analyses of stabilized rice bran, goat meat, and chili seasoning are presented in Table 1. The proximate analysis percentages for rice bran are similar to those found in the literature [42], but the percentages for goat meat analysis differed from those obtained by Devendra [8]. Goat meat moisture and protein percentages were lower than those reported by Devendra, while fat percent was higher and ash percent was similar [8]. According to Devendra, goat meat had lower fat content than fat content in this study because in their study the tissue was more concentrated in viscera than the muscle. In this study, a mixture of shoulder, leg, and other parts of meats of one-to-three-year-old goats was used, while Devendra used meat from kids (young goat). The composition of meat depends on the age of animal and the part of the animal used [43].

The proximate analyses of sausages with added rice bran are presented in Table 2. The moisture content of the raw sausages decreased slightly in response to increased rice bran percentage. In contrast, the moisture content of cooked sausages decreased linearly in response to increasing rice bran percentages. This was due to the addition of ample water to the mixture before rice bran is added at three different levels and 6-7 hours of cooking and smoking process. Also, the results of this study for cooked goat meat sausage are similar to other studies [6]. The percentages of fat, ash and protein were greater in the cooked sausages than the raw sausages. This probably was due to the moisture loss during the treatment and cooking process. The fat content of both raw and cooked sausages increased linearly in response to increasing rice bran percentages as a result of addition of fat from rice bran. Thus, sausages with added rice bran might have a more beneficial fatty acid composition in regard to more mono- and poly unsaturated fatty acids [15] as discussed in Section 3.2. The ash percent of raw sausages did not vary significantly across the rice bran percentage, The ash percent of cooked sausages decreased linearly in response to the increasing rice bran percentage, even though the variation among means was small. This could be due to a very sensitive equipment Phoenix microwave furnace (CEM corp., Matthews, NC), the variability within the replicates were so small that significant differences (*P* < 0.001) were detected. There was no significant effect of added rice bran on the protein content of raw sausages and cooked sausages. On the other hand, the cooking and smoking process increased the percentage of protein. The carbohydrate percentage of raw and cooked sausages did not changed significantly in response to the increasing rice bran percentages. This could be due to cooking and smoking process. Cooking process decreased the carbohydrate percentage from raw to cook. In this study we did not measure cooking yield due to complexity of the cooking and smoking process.

### 3.2. Fatty Acids

Fatty acids are important components of meat lipids [10] which can have an important influence on human plasma cholesterol level. For instance, saturated fatty acids increase cholesterol levels in contrast to total monounsaturated fatty acids (MUFA) and total polyunsaturated fatty acids (PUFA). Not all saturated fatty acids have the same influence on the cholesterol level; C16:0 and C14:0 can increase cholesterol levels, while C18:0 does not have such effect [10].

Fatty acid content, as percentages of total fatty acids, for goat meat reported in the literature are oleic acid (C18:1) 28–50%, palmitic (C16:0) 15–31%, stearic (C18:0) 6–17%, and linoleic (C18:2) 4–15% [10]. The high concentration of C18:0 in goat meat will not increase cholesterol levels in human, and more importantly, the ratio of (C18:0 + C18:1) to C16:0 could have beneficial health effects when it falls between 2 and 3 [10]. Meat can be also classified to have health benefits by concentration of desirable fatty acids (DFA) which include the total of C18:0 and all unsaturated fatty acids. These DFA are considered to have either neutral or cholesterol lowering effects [10].

Essential omega-6 and omega-3 fatty acids play a crucial role in the human body. They carry fat soluble vitamins and support immune systems. These fatty acids cannot be synthesized by human organisms and need to be consumed in the diet [35]. The C20:4 eicosatetranoic fatty acid is an essential fatty acid [10] which can be derived either from C18:2 linoleic fatty acids or directly from the diet. Goat muscle contains nearly twice as much C20:4 as lamb muscle [10]. The C18:2 and C20:4 fatty acid contents in goat meat used for this study were lower than in the literature which may be explained by the utilization of different parts of the goat and older goats that contain less total PUFA.

The fatty acid compositions of cooked goat meat sausages with added rice bran are presented in Table 3. Goat meat, stabilized rice bran, and chili seasoning were analyzed for fatty acid composition also (data not shown). The fatty acid results for rice bran were comparable to previously published results [36]. The fatty acids composition of goat meat sausages without rice bran was comparable to goat patties without oat bran [6]. The individual fatty acid components and sums and ratios of individual fatty acids are presented as a percentage of total fatty acids. The percentage of total fatty acids of 5 of the 12 individual fatty acids increased linearly in response to the increasing rice bran percentages, while only 2 fatty acids decreased with increased rice bran. The percentage of total fatty acids for the sum of the saturated fatty acids (∑Sat) in unmixed rice bran was 22.7% (data not shown) compared to 65.2% for goat meat sausage without rice bran as shown in Table 3. Consequently, it was expected that incorporation of rice bran to decrease the saturated fatty acids content of goat meat sausages. In this study, the total saturated fatty acids decreased significantly (*P* < 0.001) in response to increasing rice bran percentage of the goat meat sausages (Table 3). The percentage of total fatty acids for the ∑MUFA in unmixed rice bran was 49.8% (data not shown) compared to 30.7% in goat meat sausages without rice bran as shown in Table 3. Therefore, the incorporation of rice bran, into goat meat sausages was expected to increase the ∑MUFA of the goat meat sausages. In this study, the percentage of total fatty acids for ∑MUFA increased significantly (*P* < 0.05) in response to increasing rice bran percentages of the goat meat sausages (Table 3). The percentage of the total fatty acids of the monounsaturated fatty acids C16:1 and C18:1 increased linearly. The percentage of total fatty acids for 3 of the 7 polyunsaturated fatty acids C18:2n6, C20:2n6, and C20:5n3 increased linearly in response to increasing rice bran. In contrast, the percentage of total fatty acids of one polyunsaturated fatty acid C20:3n6 decreased linearly.

The (C18:0 + C18:1) to C16:0 ratio did not vary significantly in response to the increasing rice bran percentages. Thus, the (C18:0 + C18:1) to C16:0 ratio remained in the healthy range of 2-3 [10]. In this study, the ratio was from 2.7–2.9 across the rice bran percentages, which is in agreement with reported values [10]. The percentage of total saturated (∑Sat) decreased linearly with increasing rice bran. The percentage of total MUFA and total PUFA increased linearly with increasing rice bran from 30.7% at zero percent rice bran to 33.4% at 3% rice bran. Subsequently, the percentage of total PUFA increased from 4.3% at zero percent rice bran to 5.2% at 3% rice bran. The percentage of total omega-6 and total omega-3 in sausages increased linearly with increased rice bran from 3.97% and 0.31% at zero percent rice bran to 4.64 and 0.51% at 3% rice bran, respectively. These differences were significant (*P* < 0.05). The omega-3 to omega-6 fatty acid ratio (n3/n6) did not change significantly. The total DFA concentrations in this study increased linearly from %73.2 to %74.5 at zero percent rice bran to 3% of rice bran, respectively, which was not significant. These data are similar to data range presented by Banskalieva et al. [10]. In this study, the ratio of ∑PUFA/∑Sat was lower than in some reported literature [10] and similar to others [44]. The ratio of ∑PUFA/∑Sat increased linearly with increasing rice bran at a significant level (*P* < 0.001). This could be an additional health benefit of incorporating rice bran in the sausages. There was a linear increase in the fat percent of sausages in response to increasing rice bran percentages (Table 2). However, the percent of total saturated fatty acids decreased in response to increasing rice bran percentages of the sausages. The percentage of total unsaturated fatty acids, both MUFA and PUFA, and both total omega-6 and omega-3, increased in response to increasing rice bran percentages.

### 3.3. *α*-Tocopherol and Cholesterol Concentrations and Antioxidant Activity

The *α*-tocopherol concentrations of rice bran and cooked sausages with added rice bran are presented in Table 4. The concentration of *α*-tocopherol in rice bran was 7.37 mg/100 g which is similar to that presented in Gerhardt and Gallo [30] and Zigoneanu et al. [26]. The concentration of *α*-tocopherol increased linearly in response to the increasing rice bran percentages of the sausages. High temperatures can cause *α*-tocopherol degradation, but a previous study has shown no significant degradation with the cooking conditions used in this experiment [45]. The cholesterol concentration did not vary significantly in response to the increasing rice bran percentages. The antioxidant activity of unmixed rice bran and goat meat were 237.0 and 5.1 moles of Trolox equivalent per 100 g, respectively. The antioxidant activity increased linearly from 13.5 to 22.7 moles of Trolox equivalent/100 g in response to increasing rice bran percentages of the sausages (Table 4).

## 4. Conclusions

Goat meat sausages with 3% of stabilized rice bran had higher concentration of *α*-tocopherol, higher antioxidant activity, higher total omega 3/omega 6 ratio, higher total MUFA and total PUFA content, nonsignificant higher concentration of DFA, and an acceptable ratio of (C18:0 + C18:1) to C16:0 compared to zero percent rice bran. The (C18:0 + C18:1) to C16:0 ratio remained in the healthy range of 2 to 3 [10] across the rice bran percentages. Thus, sausages with added rice bran have a more beneficial fatty acid composition in regard to more mono- and polyunsaturated fatty acids as discussed in Section 3.2. Based on the results from this study and because of these factors, adding 3% stabilized rice bran to goat meat sausage is recommended which will provide more health benefits.

## Figures and Tables

**Table 1:** Proximate composition of stabilized rice bran, goat meat, and chili seasoning (Means ± s.e.).

Proximate analysis	Stabilized rice bran	Goat meat	Chili seasoning
	(% by weight)		
Moisture	18.4 ± 0.08	70.8 ± 0.20	4.3 ± 0.50
Fat	15.6 ± 0.10	10.9 ± 0.46	5.2 ± 0.01
Ash	9.6 ± 0.03	0.9 ± 0.01	13.1 ± 0.04
Protein	11.5 ± 0.18	14.6 ± 0.78	10.0 ± 0.08
Carbohydrates	45.1 ± 0.03	2.8 ± 0.15	66.3 ± 0.50

**(a):** 

Proximate analysis	Raw				
	Percent rice bran				
	0	1.5	3	Mean^†^	Linear effect^‡^
	(% by weight)				
Moisture^#^	72.2^a^ ± 0.009	70.9^a^ ± 0.09	70.1^a^ ± 0.04	71.1 ± 0.31	−∗∗∗
Fat^#^	8.9^b^ ± 0.28	9.7^b^ ± 0.35	10.5^b^ ± 0.21	9.7 ± 0.27	+∗∗∗
Ash	1.5 ± 0.01	1.5 ± 0.01	1.5 ± 0.02	1.5^B^ ± 0.01	ns
Protein	13.1 ± 1.29	13.3 ± 2.05	11.8 ± 1.06	12.8^B^ ± 0.80	ns
Carbohydrates	4.3 ± 1.14	4.6 ± 2.08	6.1 ± 1.32	5.0^A^ ± 0.83	ns

**(b):** 

Proximate analysis	Cooked				
	Percent rice bran				
	0	1.5	3	Mean^†^	Linear effect^‡^
	(% by weight)				
Moisture^#^	63.6^b^ ± 0.50	62.9^b^ ± 0.13	58.3^b^ ± 0.04	61.6 ± 0.86	−∗∗∗
Fat^#^	13.1^a^ ± 0.03	12.3^a^ ± 0.06	15.1^a^ ± 0.20	13.5 ± 0.42	+∗∗∗
Ash	2.1 ± 0.01	2.0 ± 0.02	2.0 ± 0.02	2.0^A^ ± 0.02	−∗∗∗
Protein	20.5 ± 1.00	21.1 ± 0.20	21.6 ± 0.32	21.1^A^ ± 0.34	ns
Carbohydrates	1.0 ± 1.00	1.7 ± 0.40	3.0 ± 0.56	1.8^B^ ± 0.46	ns

^§^All sausages contain chili seasoning (3% by weight) and salt (1.6% by weight).

^†^Different upper case letters (A and B) following a raw and cooked mean (across percent rice bran levels) indicates that those two means differ (*P* < 0.05).

^#^Moisture and Fat exhibited a significant preparation by percent rice bran interaction; therefore raw and cooked means were not compared. Different lower case letters (a and b) following a percent rice bran mean (within a percent rice bran level) indicates that raw and cooked differ (*P* < 0.05).

^‡^The − sign indicates a negative response and the + sign a positive response to increasing percent rice bran levels.

^‡∗, ∗∗, ∗∗∗^ indicates the linear effect, significant at the 0.05, 0.01, 0.001 level of probability, respectively. ns: not significant.

**Table 3:** Fatty acid composition of cooked goat meat sausages with added rice bran (Means ± s.e.)^§^.

Fatty acids	Percent rice bran			Linear effect^‡^
	0.0	1.5	3.0	
Saturated fatty acids				
C14:0	2.37 ± 0.04	2.48 ± 0.01	2.28 ± 0.03	ns
C16:0	24.40 ± 0.77	23.75 ± 0.20	23.19 ± 0.11	ns
C18:0	38.25 ± 0.14	34.76 ± 0.35	36.00 ± 0.10	−∗∗∗

Mono and poly unsaturated fatty acids				
C16:1	1.17 ± 0.01	1.41 ± 0.01	1.27 ± 0.02	+∗∗
C18:1	29.51 ± 0.78	32.52 ± 0.3	32.09 ± 0.30	+∗
C18:2n6	3.02 ± 0.15	3.56 ± 0.14	3.67 ± 0.17	+∗
C18:3n6	0.27 ± 0.04	0.32 ± 0.03	0.27 ± 0.02	ns
C18:3n3	0.30 ± 0.02	0.38 ± 0.02	0.46 ± 0.10	ns
C20:2n6	0.03 ± 0.01	0.03 ± 0.02	0.06 ± 0.004	+∗
C20:3n6	0.09 ± 0.01	0.07 ± 0.01	0.00 ± 0.00	−∗∗∗
C20:4n6	0.57 ± 0.06	0.65 ± 0.02	0.63 ± 0.03	ns
C20:5n3	0.01 ± 0.00	0.06 ± 0.03	0.06 ± 0.01	+∗∗

Sums and ratios of fatty acids				
∑Sat	65.02 ± 0.79	61.00 ± 0.31	61.48 ± 0.20	−∗∗
∑MUFA	30.69 ± 0.77	33.93 ± 0.32	33.36 ± 0.30	+∗
∑PUFA	4.28 ± 0.15	5.07 ± 0.08	5.15 ± 0.23	+∗∗
DFA	73.2 ± 0.80	73.6 ± 0.19	74.5 ± 0.12	ns
(C18:0 + C18:1)/C16:0	2.78 ± 0.15	2.83 ± 0.04	2.94 ± 0.02	ns
∑n6	3.97 ± 0.02	4.63 ± 0.09	4.64 ± 0.20	+∗
∑n3	0.31 ± 0.02	0.44 ± 0.02	0.51 ± 0.09	+∗
∑n3/∑n6	0.08 ± 0.00	0.09 ± 0.00	0.11 ± 0.02	ns
∑PUFA/∑Sat	0.06 ± 0.01	0.08 ± 0.00	0.08 ± 0.00	+∗∗

^§^All sausages contain chili seasoning (3% by weight) and salt (1.6% by weight).

^‡^The − sign indicates a negative response and the + sign a positive response to increasing percent rice bran levels.

^‡^Indicates the linear effect, ^∗,∗∗,∗∗∗^Significant at the 0.05, 0.01, 0.001 level of probability, respectively.

∑Sat = sum of saturated fatty acids; ∑MUFA = sum of monounsaturated fatty acids; ∑PUFA = sum of polyunsaturated fatty acids.

DFA (Desired fatty acids) = C18:0 + ∑MUFA + ∑PUFA; ∑n6 = sum of omega-6 fatty acids; ∑n3 = sum of omega-3 fatty acids. ns: not significant.

**Table 4:** *α*-Tocopherol, cholesterol, and antioxidant activity of rice bran and cooked goat meat sausages with added rice bran (Means ± s.e.)^§^.

		Goat meat sausage			
	Rice bran	Percent rice bran			Linear effect^‡^
		0	1.5	3	
		(mg/100 g)			
*α*-Tocopherol	7.37 ± 0.21	0.96 ± 0.08	0.99 ± 0.08	1.40 ± 0.02	+∗∗
Cholesterol		51.4 ± 0.90	51.4 ± 1.78	51.0 ± 2.18	ns

		(*μ*moles of Trolox equivalent/100 g)			
Antioxidant activity	236.95 ± 0.26	13.5 ± 0.38	14.3 ± 0.005	22.7 ± 0.02	+∗∗∗

^§^All sausages contain chili seasoning (3% by weight) and salt (1.6% by weight).

^‡^The + sign indicates a positive response to increasing percent rice bran levels.

^‡^Indicates the linear effect, ^∗,∗∗,∗∗∗^Significant at the 0.05, 0.01, 0.001 level of probability, respectively.

ns: not significant.
